# Immigration, language education, & trauma: exploring the intersectionality of gay Dominican immigrant experiences

**DOI:** 10.3389/fsoc.2025.1438408

**Published:** 2025-08-08

**Authors:** Jordan González

**Affiliations:** St. John's University, New York, NY, United States

**Keywords:** intersectionality, LGBTQ+ immigrants, Dominican immigrants, trauma, language education, resilience, New York City

## Abstract

This article explores the intersectionality of immigration, language education, and trauma among gay Dominican immigrant men living in New York City. Utilizing a qualitative case study approach, the research examines the lived experiences of four individuals, highlighting the compounded adversities they face due to their intersecting identities as LGBTQ+, men of color, and immigrants. The case studies reveal significant trauma and mental health struggles stemming from pre-migration violence, in-transit dangers, and post-migration discrimination. Additionally, the study addresses the critical role of language proficiency in their integration and the importance of tailored support systems, including community networks and legal protections. The findings underscore the severe impact of discrimination based on sexual orientation, race, and immigration status on the mental health and social integration of gay Dominican immigrant men. Despite these challenges, the participants demonstrate remarkable resilience and hope for the future, striving for better employment opportunities, educational advancement, and community belonging. This article provides insights into the specific needs of LGBTQ+ immigrants and offers recommendations for improving support systems, policies, and educational practices to better serve this vulnerable population.

## Introduction

The intersectionality of immigration, language education, and trauma presents a complex and multifaceted challenge, particularly for queer immigrants from marginalized communities. This article focuses on the unique experiences of gay Dominican immigrant men living in New York City, exploring how their intersecting identities impact their lives. Queer immigrants face compounded adversities, including discrimination based on their sexual orientation, race, language proficiency, and immigration status, which significantly affect their mental health and ability to integrate into their new environment (Attia et al., 2023; Rodriguez and Xiong, 2019). For gay Dominican immigrants in New York City, the challenges are particularly acute. They must navigate the complexities of being a racial minority, an immigrant, and a member of the LGBTQ+ community simultaneously.

This article employs a qualitative study approach, specifically utilizing case studies, to explore the themes that emerge from the lived experiences of gay Dominican immigrants. A case study approach allows for an in-depth examination of individual lived experiences, revealing details, and providing a nuanced understanding of how intersecting identities shape the lives of LGBTQ+ immigrant groups (Comas-Díaz, 2021). Through detailed case studies of four individuals, this article highlights their struggles, resilience, and hopes for the future. By examining their experiences through the lens of intersectionality, the study aims to provide a deeper understanding of the specific needs of LGBTQ+ immigrants and offer recommendations for better support systems, policies, and educational practices. The article also seeks to highlight the importance of language education in empowering immigrants and facilitating their integration into their new environment.

Although the broader literature discusses LGBTQ+ immigrant populations, the participants in this study all identified as gay men. Therefore, the findings specifically reflect the intersectional experiences of gay Dominican immigrant men in New York City. This reflects the limitations of a case study approach, often being suggested that it is too particular; however, case studies are valuable since a scientific discipline without a large number of thoroughly executed cases studies is a discipline without systematic production of exemplars, and a discipline without exemplars is an ineffective one (Flyvbjerg, 2006).

## Background and context

The intersectional framework is critical in understanding the compounded experiences of marginalized individuals. Intersectionality, originally conceptualized to address the overlapping oppressions faced by Black women (Crenshaw, 1989), has been applied to various contexts (Romero, 2023), including LGBTQ+ immigrants (Rodriguez and Xiong, 2019; Comas-Díaz, 2021) and immigrant and refugee trauma (Critelli and Yalim, 2023), to highlight how multiple social identities (race, gender, sexual orientation, etc.) intersect to shape unique experiences of privilege and oppression. LGBTQ+ immigrants, particularly those from Latinx backgrounds, face significant barriers that are often overlooked. Studies have shown that LGBTQ+ youth, immigrants, and refugees each face substantial challenges, which are exacerbated when these identities overlap (Rodriguez and Xiong, 2019; Skinta and Nakamura, 2021). These individuals often encounter higher rates of mental health issues, including anxiety, depression, and suicidal ideation, due to the compounded trauma experienced throughout their migration journey and settlement in a new country (Awad et al., 2021).

Lesbian, gay, and bisexual (LGB) populations experience higher rates of mental health disorders than their heterosexual counterparts—not due to their sexual orientation itself, but as a result of minority stress stemming from prejudice, discrimination, and social stigma (Meyer, 1995, 2003). Meyer's (2003) Minority Stress Model accounts for this elevated risk by identifying three key components: *distal stressors*, which are external and objective events such as discrimination and violence; *proximal stressors*, which involve internal psychological processes like expectations of rejection, concealment of sexual identity, and internalized homophobia; and *coping and resilience*, which refer to the development of adaptive strategies such as community support and identity pride that help buffer the negative effects of minority stress. Minority stress from LGBTQ+ identities are further compounded by traumatogenic stages within immigration.

## Immigration and trauma

One of the most pressing issues for LGBTQ+ immigrants is the pervasive trauma they face. This trauma can occur at various stages: pre-migration, during the journey, and post-migration (Midgette and González, 2023; Foster, 2001). Many flee their home countries to escape homophobic violence and persecution, only to encounter additional stressors such as acculturative stress, fear of deportation, and difficulties in accessing education and stable employment upon arrival in the United States??. Trauma may occur in each migration stage or across stages. These challenges are further compounded by the barriers related to language proficiency and the need for effective language education.

Recent research extends Meyer's Minority Stress Model to highlight the unique, compounded stressors that LGBTQ+ immigrants and refugees face when multiple marginalized identities intersect. For example, Alessi et al. (2020) conducted a qualitative study of LGBTQ+ refugees from Muslim-majority countries who resettled in Austria and the Netherlands, revealing that participants encountered multiple, layered forms of discrimination based on sexual orientation, gender identity, race, religion, and immigration status. The study found that Islamophobic attitudes in host countries and prejudice from their own diaspora communities left many LGBTQ+ refugees socially isolated and dependent on host community members who themselves sometimes perpetuated discrimination. These findings underscore how distal stressors—such as overt xenophobia and subtle exclusion—and proximal stressors—like internalized stigma and fear of rejection—compound to heighten psychological distress, consistent with Meyer's (1995, 2003) model. This work illustrates the importance of situating queer immigrant and refugee experiences within an intersectional minority stress framework to better understand the integration barriers they face and to inform more inclusive support systems (Alessi et al., 2020).

## Cumulative racial/ethnic trauma

The concept of cumulative racial/ethnic trauma provides a framework for understanding the layered and ongoing trauma faced by immigrants due to historical, national, and institutional discrimination. This framework is particularly relevant for racial minority immigrants, such as those from the Middle Eastern and North African (MENA) regions, who experience trauma before, during, and after migration (Awad et al., 2021). The trauma often begins with the conditions in their home countries, including violence, political strife, and oppression, and continues with the acculturative stress and discrimination they face in their host countries (Awad et al., 2021).

In the United States, Ramirez and Paz Galupo (2019) build on Meyer's Minority Stress Model by examining how multiple minority stress processes affect the mental health of lesbian, gay, and bisexual people of color (LGB-POC). Their study found that both distal stressors—like microaggressions and daily heterosexist experiences—and proximal stressors—such as internalized stigma, sexual orientation rumination, and identity salience—significantly predicted depression and anxiety symptoms. Notably, their regression analyses showed that proximal stressors accounted for an additional 15% of the variance in mental health outcomes beyond what distal stressors explained alone, highlighting the substantial psychological toll of internalized oppression. Furthermore, bisexual participants reported even higher levels of distress compared to monosexual participants, suggesting that intersecting sexual, racial, and, potentially, immigration-related minority stressors can combine in unique and harmful ways (Ramirez and Paz Galupo, 2019; White et al., 2024). This study reinforces the need to apply an intersectional perspective when examining the mental health and resilience of Latinx and other LGBTQ+ people of color, aligning with the themes emerging from this case study of gay Dominican immigrant men.

## The role of language education

Language proficiency is a critical factor in the integration process for immigrants (Schuss, 2018). For LGBTQ+ immigrants, mastering the English language is not only essential for better employment opportunities but also for advocating for their rights and accessing necessary services. However, learning a new language while dealing with the trauma of migration and discrimination can be an overwhelming task (Skinta and Nakamura, 2021). Effective language education, tailored to the unique needs of LGBTQ+ immigrants, is crucial in helping them navigate their new environment and build a stable, fulfilling life (Rodriguez and Xiong, 2019).

## Theoretical frameworks

To complement the understanding of intersectionality specifically to Latinx LGBTQ+ immigrant populations, this article utilizes several theoretical frameworks to understand the experiences of gay Dominican immigrant men living in New York City. The cumulative disadvantage theory explains how prolonged exposure to various forms of disadvantage and discrimination leads to significant negative outcomes over time (Burgess, 2021). The minority stress model further explains the mental health challenges faced by LGBTQ+ individuals due to the chronic stress from their marginalized status (Skinta and Nakamura, 2021; Meyer, 2003, 1995). Finally, queer migration theory helps to contextualize the specific experiences of LGBTQ+ immigrants, highlighting how their sexual orientation and gender identity intersect with their migration status to create unique challenges and experiences (Rodriguez and Xiong, 2019). These frameworks collectively provide a comprehensive understanding of the multifaceted challenges faced by gay Dominican immigrant men in New York City. By examining their experiences through these lenses, this article aims to shed light on the specific needs of LGBTQ+ immigrants and offer insights into how support systems, policies, and educational practices can be improved to better serve this vulnerable population.

## Methodology

### Research design

This study employs a qualitative approach using case studies to explore the intersectionality of immigration, language education, and trauma among LGBTQ+ Dominican immigrants in New York City. The case study approach is particularly suited for in-depth exploration of complex social phenomena within real-life contexts (González, 2021; Yin, 2014). By focusing on individual experiences, this approach allows for a detailed understanding of how intersecting identities impact the lives of queer immigrants.

### Participants

The study focuses on four LGBTQ+ Dominican immigrants in New York City, aged between 24 and 32. The four participants identified themselves as men and either “gay” or “homosexual.” They have varying documentation statuses, including undocumented, asylum seeking, student visa, and visa sponsored. The selection of participants aimed to capture a range of experiences related to immigration status, educational background, and employment. Table 1 below displays the participant demographic data. Informed consent was obtained from all participants, and their confidentiality and anonymity were ensured through the use of pseudonyms. Data were securely stored, and participants were assured that their participation was voluntary and that they could withdraw at any time without any repercussions.

**Table 1:** Participant demographics.

**Participant**	**Age**	**Immigration status**	**Education**	**Occupation**
Carlos Peralta	27	Seeking Asylum	Secondary School	Independent Barber
Juan Martinez	29	Student Visa	College	Works in a cellular phone store
Miguel Rodriguez	24	Undocumented	Elementary School	Works in a grocery store
Luis Torres	32	Resident	College	Hospital Translator

### Data collection and participant recruitment

Data collection involved the use of semi-structured interviews. The interviews explored participants' backgrounds, immigration experiences, experiences with trauma, education experiences, work experiences, English language learning, and their hopes and aspirations for the future. Participants were given the option to respond in the language they preferred (Spanish or English). The interviews took place in-person, recorded, and transcribed.

Participants for this study were recruited through convenience sampling by accessing informal networks of gay Dominican immigrants living in New York City. Initial contacts were made through local LGBTQ+ community organizations and social groups, with participants referring one another to the researcher through word-of-mouth and trusted connections. This snowball-like referral process ensured access to individuals who were willing to share their personal migration and identity narratives. Participants were selected based on the relevance and depth of the stories they shared during preliminary conversations, and only the data from the in-depth, semi-structured interviews conducted after selection were included in the final analysis. This research project received ethics approval from the author's university Institutional Review Board (IRB) on 5 August 2024, ensuring that all procedures complied with ethical guidelines for confidentiality, informed consent, and voluntary participation.

### Analysis

Data analysis involved two cycles of coding. The first cycle focused on labeling data chunks to identify patterns and themes within each case. The second cycle involved cross-case comparisons to uncover common themes and variations across cases (Yin, 2014). Initial coding identified key themes related to trauma, language learning, and integration. Axial coding was used to explore relationships between themes and develop a comprehensive understanding of participants' experiences (Saldaña, 2016). Common themes included trauma and mental health struggles, intersectionality of identities, employment and economic hardship, language barriers and educational pursuits, support systems and community, and resilience and hope for the future. These themes are discussed in the subsequent sections.

### Case studies of gay dominican immigrant men in New York City

To gain a deeper understanding of the unique challenges and resilience of gay Dominican immigrant men in New York City, this section presents detailed case studies of four individuals: Carlos Peralta, Juan Martinez, Miguel Rodriguez, and Luis Torres. These case studies illuminate how intersecting identities—such as being part of the LGBTQ+ community, men of color, and immigrants—shape their experiences. Each narrative highlights the personal struggles, employment circumstances, educational backgrounds, language proficiency, and coping mechanisms of these individuals.

The case studies explore their varied documentation statuses, ranging from undocumented to asylum seeking, and their diverse educational achievements, from high school dropout to college graduate. Through their stories, we examine the compounded adversities they face, including discrimination, economic hardship, and mental health challenges, as well as their remarkable resilience and hope for the future. These narratives provide valuable insights into the lived experiences of LGBTQ+ immigrants and underscore the importance of tailored support systems and policies to aid their integration and wellbeing. By focusing on individual stories, this section aims to provide a comprehensive and nuanced understanding of how intersecting identities impact the lives of gay Dominican immigrant men in New York City, shedding light on common themes and patterns across cases.

### Carlos Peralta

Carlos Peralta, a 27-year-old gay man from Santo Domingo, fled the Dominican Republic less than a year ago to escape homophobic violence. Growing up, Carlos faced severe bullying and physical abuse in school due to his sexual orientation. His family's initial support waned when they became targets of community ostracism. Seeking safety, Carlos embarked on a perilous journey to the United States by flying into Guatemala, traveling into Mexico, and then crossing the U.S. Mexican Border. During the crossing, he turned himself in to border patrol indicating he is seeking asylum to escape violence in the Dominican Republic due to his sexuality.

Carlos's asylum application is pending, leaving him in a state of uncertainty. Despite his high school education, his lack of documentation limits his job prospects. He works long hours as a barber, a job that barely covers his living expenses. Carlos struggles with the trauma of his past and the fear of deportation. Determined to improve his English, Carlos enrolled in evening classes at a local community center. Learning English has been a challenge due to his demanding job and constant anxiety. However, he remains committed, knowing that better language skills could open doors to more opportunities.

Carlos finds solace in the small but supportive LGBTQ+ community in his neighborhood. They provide a sense of belonging and help him cope with his ongoing struggles. His dream is to complete his asylum process successfully and eventually pursue further education in the U.S.

### Juan Martinez

Juan Martinez, 29, from Santiago, arrived in New York City on a student visa to enroll in English language training program. Juan, who is openly gay, faced societal discrimination and familial rejection in the Dominican Republic. His decision to study in the U.S. was also an escape from his family as well as seeking economic opportunities.

Juan's advanced English proficiency helped him secure a job in a cellular phone store front; however, his pay in unreported due to not having permission to work since he is on a student visa. Interestingly Juan admits that he doesn't attend the English language training program but maintains matriculation to sustain his student visa. However, his visa is set to expire, and he plans to remain in the country undocumented unless he can find someone to marry him, either for love or by paying someone ten-thousand dollars.

The pressures of maintaining his visa status adds to his anxiety of working long hours and paying rent. His outlet from these daily pressures is a social group of gay Dominican men that keep communication using the telecommunication application “WhatsApp” and going to local bars, both gay and heteronormative, together. He hopes he can remain in the U.S. and gain permission to work so that he can secure a job that will provide him with economic and social mobility.

### Miguel Rodriguez

Miguel Rodriguez, 24, left the Dominican Republic 6 years ago, escaping an environment of severe homophobia and economic hardship. With limited education and no high school diploma, Miguel's options were limited. His undocumented status further complicates his situation, as he lives in constant fear of being deported.

Miguel's basic English proficiency restricts his job opportunities. He works at a convenience store, where he faces long hours and low pay. The language barrier makes it difficult for him to advocate for himself or seek better employment. He often feels isolated and struggles to communicate effectively with customers and coworkers. The trauma of his journey to the U.S. remains fresh in Miguel's mind. He endured dangerous conditions and exploitation during his migration, experiences that have left deep psychological and physical scars. While crossing the U.S.-Mexico border, his right arm and leg got caught on barbed wires when running across a river hearing gun shots in the air. In New York, he continues to face discrimination, both for his undocumented status and his sexual orientation.

Miguel attends free English classes whenever possible, but his progress is slow due to his demanding work schedule. Despite these challenges, he dreams of 1 day obtaining legal status, improving his English, and pursuing further education. The support from a local LGBTQ+ organization provides him with some hope and community, helping him survive in the tough environment of NYC.

### Luis Torres

Luis Torres, 32, came to New York City 13 years ago on a family-based immigrant visa sponsored by his mother and currently holds U.S. residency. With a college degree from the Dominican Republic and a high fluency in English, Luis found work as a translator in a hospital. His work is both challenging and fulfilling, allowing him to use his language skills and education to help others. However, his journey was not without its challenges.

Growing up as a gay man in a conservative neighborhood in Moca, Luis faced constant pressure to conform. His decision to move to the U.S. was driven by both socio-economic aspirations and the need for personal freedom. While his job provides financial stability and a supportive work environment, Luis continues to grapple with the trauma of his past. Luis's fluency in English has been a significant asset, allowing him to excel in his job and build a network of friends and colleagues. However, the memories of his adolescence include meetings with men for anonymous encounters, and in one instance his life was threaten with a knife. His assailant forcing him to hand over his cash and cellular phone. Despite his professional success, Luis struggles with the trauma of his past. The memories of his adolescence haunt him, making it difficult to form intimate relationships. He attends therapy sessions, which help him navigate his mental health challenges.

No longer feeling the need to hide his identity, Luis volunteers with local LGBTQ+ groups, offering support and guidance to other immigrants facing similar struggles. His experience has made him passionate about advocacy and helping others navigate the complexities of life as an LGBTQ+ immigrant. His goal is to obtain U.S. citizenship and continue his work in both the hospital and advocating for LGBTQ+ rights and immigrant support. His journey is a testament to his resilience and commitment to making a difference despite his personal hardships.

## Discussion

### Themes

The analysis of the case studies of gay Dominican immigrant men in New York City revealed several common themes that highlight the intersectional challenges and resilience of this population. These themes include compounded trauma and mental health struggles; the intersectionality of identities; employment, economic hardship, and language barriers; and community support systems, resilience, and hope.

#### Compounded trauma and mental health struggles

Each individual case study revealed significant trauma experiences that occurred at various stages of migration—pre-migration, in transit, and post-migration—demonstrating the layers of adversity that shape the mental health of gay Dominican immigrant men. For example, Carlos Peralta fled Santo Domingo after enduring severe bullying, physical violence, and community ostracism due to his sexual orientation, only to face a dangerous journey through Guatemala and Mexico, where he risked his life to cross the border. Miguel Rodriguez also recounted harrowing memories of his migration journey; while crossing the U.S.-Mexico border, he was physically injured by barbed wire while running to escape threats of gunfire, leaving him with lasting physical and psychological scars. Luis Torres, though he migrated through a family-sponsored visa, spoke of being threatened with a knife during his adolescence when meeting men for anonymous encounters in the Dominican Republic, a memory that continues to impact his ability to form intimate relationships. Juan Martinez described the constant fear of deportation due to his precarious student visa status, coupled with rejection from his family in Santiago. These personal accounts illustrate how pre-migration homophobic violence, familial rejection, and the inherent dangers of migration contribute to layers of trauma. Consistent with the broader literature on cumulative and minority stress (Awad et al., 2021; Rodriguez and Xiong, 2019), these compounded experiences place participants at heightened risk for severe mental health challenges such as anxiety, depression, and symptoms consistent with post-traumatic stress disorder (PTSD) (Meyer, 2003). Despite these challenges, each participant's narrative also reflects the daily struggle to manage this psychological burden while attempting to build a new life in an often-hostile host society.

#### Intersectionality of identities

The overlapping marginalized identities of being gay, men of color, and immigrants exacerbate the layers of discrimination and social exclusion experienced by these individuals. The case studies clearly illustrate how intersectionality amplifies their vulnerability in ways that single-axis analyses cannot capture. For instance, Carlos Peralta not only navigates the stigma of being openly gay but also faces barriers as an asylum seeker who must prove his fear of persecution while working informally as an independent barber to survive. Miguel Rodriguez's undocumented status adds another layer of precarity: as a young, undocumented gay man with limited education, he struggles to access stable employment and is forced to accept exploitative working conditions at a grocery store. Juan Martinez's story highlights how his status as a student visa holder ties him to precarious, under-the-table work while he contemplates paying someone to marry him just to remain in the country — a desperate decision shaped by the intersection of sexuality, immigrant status, and economic marginalization. Luis Torres, although documented and employed as a hospital translator, continues to confront the psychological effects of growing up gay in a conservative community in the Dominican Republic, a trauma compounded by his experiences of racialization as an immigrant man of color in the U.S. These intersecting identities generate compounded forms of minority stress, as each participant must navigate multiple systems of oppression simultaneously. This dynamic aligns with Skinta and Nakamura's (2021) assertion that intersectionality helps explain how the interplay of racial, sexual, and immigrant identities creates unique challenges more severe than those faced by individuals with only one marginalized identity.

#### Employment, economic hardship, and language barriers

The documentation status and varying levels of English proficiency among participants significantly shape their employment opportunities, economic stability, and broader integration into life in New York City. Each participant's story illustrates the stark mismatch between their aspirations, educational backgrounds, and the precarious work they are forced to accept. For example, Carlos Peralta, despite having completed secondary school, works long hours as an independent barber with an unstable income while awaiting asylum approval—a process that leaves him in limbo and vulnerable to exploitation. Miguel Rodriguez, who left the Dominican Republic with only an elementary education, remains undocumented and works for low wages at a grocery store, his limited English proficiency making it nearly impossible to negotiate better pay or advocate for himself. Juan Martinez, who arrived on a student visa, has advanced English skills yet is unable to work legally in the U.S., forcing him to take unreported jobs in a cellular phone store while balancing the stress of a visa that could expire at any moment. In contrast, Luis Torres's high level of English proficiency and college degree enabled him to secure a more stable job as a hospital translator, yet even he must navigate subtle workplace discrimination linked to his immigrant status and sexual orientation. These examples show that for gay Dominican immigrant men, documentation status and language proficiency intersect to limit access to fair employment and upward mobility. Without tailored, trauma-informed language education and pathways to legal status, these barriers perpetuate cycles of economic hardship and social exclusion, as reflected in both the individual narratives and broader literature (Schuss, 2018; Rodriguez and Xiong, 2019).

#### Community support systems, resilience, and hope

The presence of community support systems plays a vital role in helping gay Dominican immigrant men navigate the compounded adversities of homophobic violence, precarious immigration status, economic hardship, and social exclusion. Throughout the case studies, each participant demonstrates how access to LGBTQ+ communities, informal peer networks, and supportive individuals provides critical emotional support, practical resources, and a sense of belonging (Rodriguez and Xiong, 2019). For example, Carlos Peralta finds solace in a tight-knit LGBTQ+ community within his neighborhood, which offers him connection and emotional refuge while he waits for his asylum claim to be processed. Juan Martinez relies on a core group of gay Dominican friends who keep in touch through WhatsApp, sharing strategies to cope with the stress of legal liminality and social isolation. Miguel Rodriguez, despite his undocumented status and limited English, draws hope from local LGBTQ+ organizations that provide him with information about legal rights and English language classes. Luis Torres, who holds residency and stable employment, exemplifies how resilience and hope can evolve into community advocacy—he volunteers with LGBTQ+ groups to support other immigrants navigating similar challenges. Across these narratives, resilience emerges not as an individual trait alone but as a collective process grounded in community ties, peer support, and the aspiration for a better future. Despite their significant hardships, each participant continues to pursue language learning, employment opportunities, and legal pathways that will help them build stable, fulfilling lives. Understanding these layered experiences of support, resilience, and hope is essential for educators, policymakers, and mental health professionals committed to developing effective, culturally responsive strategies to serve this multiply marginalized population.

### Implications for policy and practice

The findings from the case studies of gay Dominican immigrant men in New York City highlight several critical implications for policy and practice. Addressing the unique challenges faced by this population requires comprehensive and targeted approaches in areas such as mental health support, legal protections, education, and community support.

#### Culturally responsive, trauma-informed mental health support

Mental health support for gay Dominican immigrant men—and LGBTQ+ immigrants more broadly—must be grounded in culturally sensitive, trauma-informed models that fully recognize the compounded adversities they face. As the case studies illustrate, experiences of pre-migration violence, familial rejection, dangerous transit journeys, and post-migration discrimination create layers of trauma that intersect with stressors linked to race, sexual orientation, and precarious legal status (Awad et al., 2021). To respond effectively, therapists and mental health professionals should receive specialized training on how these intersecting identities shape individual and collective mental health needs. This includes not only understanding the Minority Stress Model but also recognizing the traumatogenic stages of migration and how stigma, isolation, and discrimination compound distress (Skinta and Nakamura, 2021). Incorporating trauma-informed care principles—such as safety, trustworthiness, empowerment, and peer support—alongside cultural responsiveness can help clients process past violence and build coping strategies that honor their linguistic, cultural, and community contexts. Tailored interventions that include bilingual therapy options, partnerships with trusted community organizations, and peer support groups can further strengthen mental health services and reduce barriers to care for this marginalized population.

#### Education and language support

Education policies must focus on providing comprehensive language education programs that cater to the needs of LGBTQ+ immigrants. Improving English proficiency is crucial for their integration and economic stability. This includes developing language education programs that consider the specific needs and schedules of working immigrants can help them achieve language proficiency more effectively. These programs should also incorporate elements of cultural adaptation and resilience-building (Awad et al., 2021). This includes policies that provide access to higher education for undocumented and asylum-seeking immigrants by providing scholarships, in-state tuition rates, and financial aid options.

#### Community support and integration

Building supportive communities and networks is vital for the wellbeing and resilience of LGBTQ+ immigrants. Community organizations play a crucial role in providing social support, resources, and advocacy. Supporting community-based organizations that cater to the needs of LGBTQ+ immigrants can help provide essential services such as legal aid, mental health support, and social integration programs (Rodriguez and Xiong, 2019). Encouraging the formation of peer support networks within immigrant communities can foster a sense of belonging and mutual assistance, which are critical for mental health and resilience (Skinta and Nakamura, 2021).

#### Employment and economic opportunities

Improving access to fair employment opportunities for LGBTQ+ immigrants is essential for their economic stability and integration. Implementing job training and placement programs specifically designed for LGBTQ+ immigrants can help them secure stable and well-paying jobs that match their skills and educational backgrounds (Awad et al., 2021). Addressing the complex needs of LGBTQ+ Dominican immigrants in New York City requires a multifaceted approach that integrates mental health support, legal protections, education, community support, and economic opportunities. By implementing policies and practices that recognize and address their intersecting identities and unique challenges, we can help these individuals achieve better integration and overall well-being.

## Limitations and future research

One notable limitation of this study is its reliance on a small, qualitative case study approach. While this method allows for an in-depth, nuanced exploration of the lived experiences of gay Dominican immigrants in New York City, its findings cannot be generalized to all LGBTQ+ immigrant populations or even all gay Dominican immigrants. The highly particular nature of case studies has often been critiqued as being too context-specific to inform broader conclusions. However, as Flyvbjerg (2006) argues, case studies remain essential to building a scientific discipline; without a sufficient number of well-executed cases, a discipline lacks systematic exemplars that make theoretical insights concrete and applicable. Additionally, the sample is limited by its geographic scope and the unique sociopolitical context of New York City, which may offer different resources and challenges than other regions. Future studies should remain mindful of these contextual factors when interpreting or applying these findings.

Building on this study's insights, future research should expand to include a larger and more diverse sample of LGBTQ+ immigrants from different cultural backgrounds, gender identities, and immigration statuses to capture broader patterns and experiences. Longitudinal studies could provide deeper understanding of how trauma, language acquisition, and resilience evolve over time as immigrants navigate changing legal and social landscapes. Comparative studies across cities or regions could reveal how local policies, community resources, and social attitudes influence the integration and wellbeing of queer immigrants. Additionally, research that directly examines the effectiveness of trauma-informed language education programs and community-based mental health interventions for LGBTQ+ immigrants would help to translate these findings into evidence-based policy and practice. Finally, participatory action research that centers the voices of queer immigrants themselves can further illuminate pathways for advocacy and systemic change.

## Conclusion

The intersectionality of immigration, language education, and trauma presents significant challenges for LGBTQ+ Dominican immigrants in New York City. This article has explored these complexities through the detailed case studies of Carlos Hernandez, Juan Martinez, Miguel Rodriguez, and Luis Torres. Their stories illustrate how compounded adversities stemming from their intersecting identities of race, sexual orientation, and immigration status profoundly impact their mental health, social integration, and economic opportunities.

The participants' narratives illustrate experiences of pre-migration violence, in-transit dangers, and post-migration discrimination, which, when considered alongside findings from the broader literature, suggest a heightened risk for severe mental health challenges such as anxiety, depression, and PTSD (Awad et al., 2021; Meyer, 2003; Rodriguez and Xiong, 2019). These narratives underscore the critical need for trauma-informed and culturally competent mental health care. The intersection of LGBTQ+ identity with racial and immigrant statuses exacerbates the discrimination and social exclusion faced by these individuals. Understanding and addressing these overlapping identities are crucial for developing effective support systems (Skinta and Nakamura, 2021).

The varied documentation statuses of the participants significantly affect their employment opportunities and economic stability. Carlos, who is seeking asylum, Miguel, who is undocumented, and Juan, who is on a student visa, face substantial barriers to securing stable and well-paying jobs. In contrast, Luis, who is a resident and traveled on a family-sponsored visa, has better employment opportunities due to their legal status and higher education. However, all participants still encounter challenges related to their LGBTQ+ identities. Addressing these disparities through policies that provide fair employment opportunities and support economic self-sufficiency is essential (Awad et al., 2021).

Language proficiency is another critical factor for integration. Carlos and Miguel struggle with significant language barriers that hinder their professional growth and daily interactions. On the other hand, Juan and Luis have advanced English skills, which help them secure better employment and navigate their new environment more effectively. Tailored language education programs that consider the specific needs of LGBTQ+ immigrants are vital for their personal and professional development (Rodriguez and Xiong, 2019).

Strong support systems, including community organizations and peer networks, play a vital role in the resilience and wellbeing of LGBTQ+ immigrants. Carlos finds solace in the local LGBTQ+ community, Juan has a core group of LGBTQ+ Dominican friends, Miguel relies on local organizations, and Luis actively volunteers with LGBTQ+ groups. These networks provide emotional support, resources, and a sense of belonging, which are crucial for their integration and mental health (Skinta and Nakamura, 2021).

Despite significant adversities, the participants demonstrate remarkable resilience and hope for the future. Carlos is determined to improve his English and pursue further education, Juan aims to achieve residency and seek better employment opportunities, Miguel wants legal status and pursue education, and Luis aspires to gain citizenship and continue his advocacy work. Their determination to improve their English, secure better employment, and build supportive communities highlights their strength and perseverance (Attia et al., 2023).

In conclusion, the narratives of Carlos, Juan, Miguel, and Luis underscore the importance of understanding the unique challenges faced by gay Dominican immigrant men through an intersectional lens. By acknowledging and addressing the specific needs of this population, policymakers, educators, and mental health professionals can develop more effective and compassionate strategies to support their integration and wellbeing. Continued research is needed to better understand the experiences of additional LGBTQ+ immigrant groups to inform policy development. Policies should focus on providing comprehensive legal protections, access to mental health care, educational opportunities, and fair employment practices. Educators and mental health professionals must receive training in cultural responsive and trauma-informed care to effectively support LGBTQ+ immigrants. Strengthening community-based organizations and peer support networks can provide critical resources and a sense of belonging for LGBTQ+ immigrants. Advocacy efforts should focus on protecting their rights and promoting social inclusion. By implementing these recommendations, we can create a more inclusive and supportive environment for LGBTQ+ Dominican immigrants and help them build stable, fulfilling lives in their new home.

## Data Availability

The datasets presented in this article are not readily available. Requests to access the datasets should be directed to Jordan Gonzalez, gonzalj6@stjohns.edu.
